# Early molecular events of Autosomal Dominant Alzheimer’s Disease in marmosets with PSEN1 mutations

**DOI:** 10.1002/alz.086608

**Published:** 2025-01-03

**Authors:** Lauren Bailey, Gregg E Homanics, Jung Eun Park, David J Schaeffer, Lauren K Hayrynen Schaeffer, Ting Zhang, Annat Haber, Catrina Spruce, Anna Greenwood, Takeshi Murai, Laura Schultz, Lauren Mongeau, Seung‐Kwon Ha, Julia Oluoch, Brianne Stein, Sang‐Ho Choi, Hasi Huhe, Amantha Thathiah, Peter L Strick, Gregory W Carter, Afonso C Silva, Stacey J Sukoff Rizzo

**Affiliations:** ^1^ University of Pittsburgh School of Medicine, Pittsburgh, PA USA; ^2^ The Jackson Laboratory for Genomic Medicine, Farmington, CT USA; ^3^ The Jackson Laboratory, Bar Harbor, ME USA; ^4^ Sage Bionetworks, Seattle, WA USA; ^5^ University of Pittsburgh, Pittsburgh, PA USA; ^6^ University of Maine, Orono, ME USA; ^7^ Tufts University, Boston, MA USA

## Abstract

**Background:**

Fundamental questions remain about the key mechanisms that initiate Alzheimer’s disease (AD) and the factors that promote its progression. Here we report the successful generation of the first genetically‐engineered marmosets that carry knock‐in (KI) point mutations in the presenilin‐1 (*PSEN1*) gene that can be studied from birth throughout lifespan.

**Method:**

CRISPR/Cas9 was used to generate marmosets with C410Y or A426P point mutations in *PSEN1*. Founders and their germline offspring are comprehensively studied longitudinally using non‐invasive measures including behavior, biomarkers, neuroimaging, and multi‐omics signatures.

**Result:**

Prior to adulthood, increases in plasma Aβ were observed in PSEN1 mutation carriers relative to non‐carriers. Analysis of brain revealed alterations in several enzyme‐substrate interactions within the gamma secretase complex prior to adulthood.

**Conclusion:**

Marmosets carrying KI point mutations in *PSEN1* provide the opportunity to study the earliest primate‐specific mechanisms that contribute to the molecular and cellular root causes of AD onset and progression.

